# The biogeography of colonial volvocine algae in the Yangtze River basin

**DOI:** 10.3389/fmicb.2023.1078081

**Published:** 2023-01-26

**Authors:** Yuxin Hu, Jing Zhang, Jie Huang, Mingchun Zhou, Sheng Hu

**Affiliations:** Yangtze River Basin Ecological Environment Monitoring and Scientific Research Center, Yangtze River Basin Ecological Environment Supervision and Administration Bureau, Ministry of Ecological and Environment, Wuhan, China

**Keywords:** colonial volvocine algae, Yangtze River basin, biogeography, metabarcoding, full-length 18S rRNA gene, random forest, MaxEnt

## Abstract

Colonial Volvocine Algae (CVA) are of great significance for biological evolution study, but little is presently known about their biogeographic distribution. Meanwhile, with the impact of climate change and human activities, their effects on the distribution and structures of CVA communities also remain largely unknown. Herein, the biogeography of CVA was investigated in the Yangtze River basin, 172 sampling sites were set up within a catchment area of 1,800,000  km^2^, and the distribution and community composition of CVA were studied using single-molecule real-time sequencing and metabarcoding technology based on the full-length 18S sequence. In 76 sampling sites, CVA was discovered in two families, eight genera, and nine species. *Eudorina* and *Colemanosphaera* were the main dominant genus. Based on the result of the random forest model and Eta-squared value, the distribution of CVA was significantly influenced by water temperature, altitude, and TP. CVA could be suitably distributed at an average water temperature of 22°C, an average TP concentration of 0.06 mg/L, and an altitude lower than 3,920  m. To assess the effects of anthropogenic pollution on the structures and co-occurrence patterns of CVA communities, we used a stress index calculated by 10 environmental factors to divide the CVA community into low and high pollution group. Network analysis showed that greater pollution levels would have a negative impact on the co-occurrence patterns and diversity of the CVA community. Finally, to study the scientific distribution of CVA under current and future climate change scenarios, we analyzed the climate suitability regionalization of CVA with the maximum entropy model based on 19 climatic factors and four climate scenarios from 2021 to 2040 published by CMIP6. Our results reveal the suitable areas of CVA, and temperature is an important environmental factor affecting the distribution of CVA. With the change of climate in the future, the Three Gorges Reservoir Area, Chaohu Lake, and Taihu Lake are still highly suitable areas for CVA, but the habitat of CVA may be fragmented, and more thorough temporal surveys and sampling of the sediment or mud are needed to investigate the fragmentation of CVA.

## 1. Introduction

The Yangtze River Basin stretches across the three major geographical regions of western, central, and eastern China. The climate spans the tropical, subtropical, and warm temperate zones, and flows through 19 provinces and cities (autonomous regions and province-level municipality) from west to east, it covers an area of approximately 1.8 × 10^6^ km^2^, accounting for 18.8% of China’s land area, it is an important ecological security barrier area in China due to its complex landform and diverse ecosystem types (Qu et al., 2018). The Yangtze River is the largest and longest river in China, its basin has elevation varying from 0 to 5,000 m with latitude from 25’N to 35’N. The Tanggula Mountains in the Tibetan Plateau’s southwest are the source of the Yangtze River’s mainstream. According to the variations in hydrology and topography, the mainstream can be classified into the following areas: the source region in Qinghai province, the Jinsha River (from Zhimenda in Qinghai province to Yibin in Sichuan province), the upper region from the Yibin city to Yichang city (Three Gorges Dam site), the middle region from Yichang city to Hukou (Poyang Lake mouth), the lower region from Hukou to Datong, and below Datong is the estuary (Wang et al., 2007). The Yangtze River has a well-developed water system, with thousands of large and small tributaries converging along the mainstream, of which the most significant are eight primary tributaries, namely, Yalong River, Minjiang River, Jialing River, Wujiang River, Hanjiang River, Yuanjiang River, Xiangjiang River, and Ganjiang River. There are also numerous lakes and reservoirs in the Yangtze River basin, all of which have ecological and social significance, including Poyang Lake, Dongting Lake, Taihu Lake, Chaohu Lake, Danjiangkou Reservoir, and the Three Gorges Reservoir. These tributaries and lakes/reservoirs have greatly enriched the diversity of the ecosystem in the Yangtze River basin and actively contributed to the social, economic, and cultural development of various regions.

Colonial volvocine algae (CVA) belong to Volvocales (Chlorophyceae and Chlorophyta), and consist of three families, Volvocaceae, Goniaceae, and Tetrabaenaceae (Desnitskiy, 2020). CVA covers all kinds of representative species from simple to complex in biological structure and reproductive mode, so it is frequently used in the study of single-cell to multicellular evolution, cell differentiation, and reproductive evolution, but its geographical distribution is much less discussed. The most recent study shows that there are at least 50 species of this group (Lindsey et al., 2021). Since more than 10 new CVA species have been discovered in recent years, additional research is still needed to uncover this group’s hidden biodiversity (Herron and Nedelcu, 2015). China is a vast country with an abundance of resources, and the varied environmental conditions must breed a variety of CVA species. However, there has been little research on the taxonomy of this group in China, China has only described less than 20 species of CVA to date, and there is still a lot of space to be explored (Hu and Wei, 2006; Yang, 2006; Hong et al., 2010; Hu et al., 2016, 2020; Hu, 2020). Meanwhile, the geographical distribution of CVA attracts attention much less often worldwide (Desnitskiy, 2020), and there is no biogeography research on CVA in China until now.

While worldwide water and temperature distribution is currently impacted by climate change, the distribution of algae is mostly constrained by factors such as water temperature, light, elevation, and CO_2_ concentration. Since the Fourth Industrial Revolution, the trend of global warming has intensified, resulting in a significant decrease in suitable habitats for some species and habitat fragmentation, putting many species in danger of extinction or already extinct, which has had a significant negative impact on ecological security and biodiversity (Basso et al., 2018; Vincent et al., 2020). Nearly one-sixth of all species in the world are currently threatened in varying degrees. Some research shows that with the rise of global temperature in the future, the risk of extinction of endangered species will greatly increase (Cao et al., 2020). Therefore, in the context of global warming, this study took the Yangtze River basin as an example to explore the geographical distribution pattern of CVA, which will contribute to the taxonomy and biogeography of CVA, thus maintaining its diversity.

## 2. Materials and method

### 2.1. Sampling

**Figure 1:**
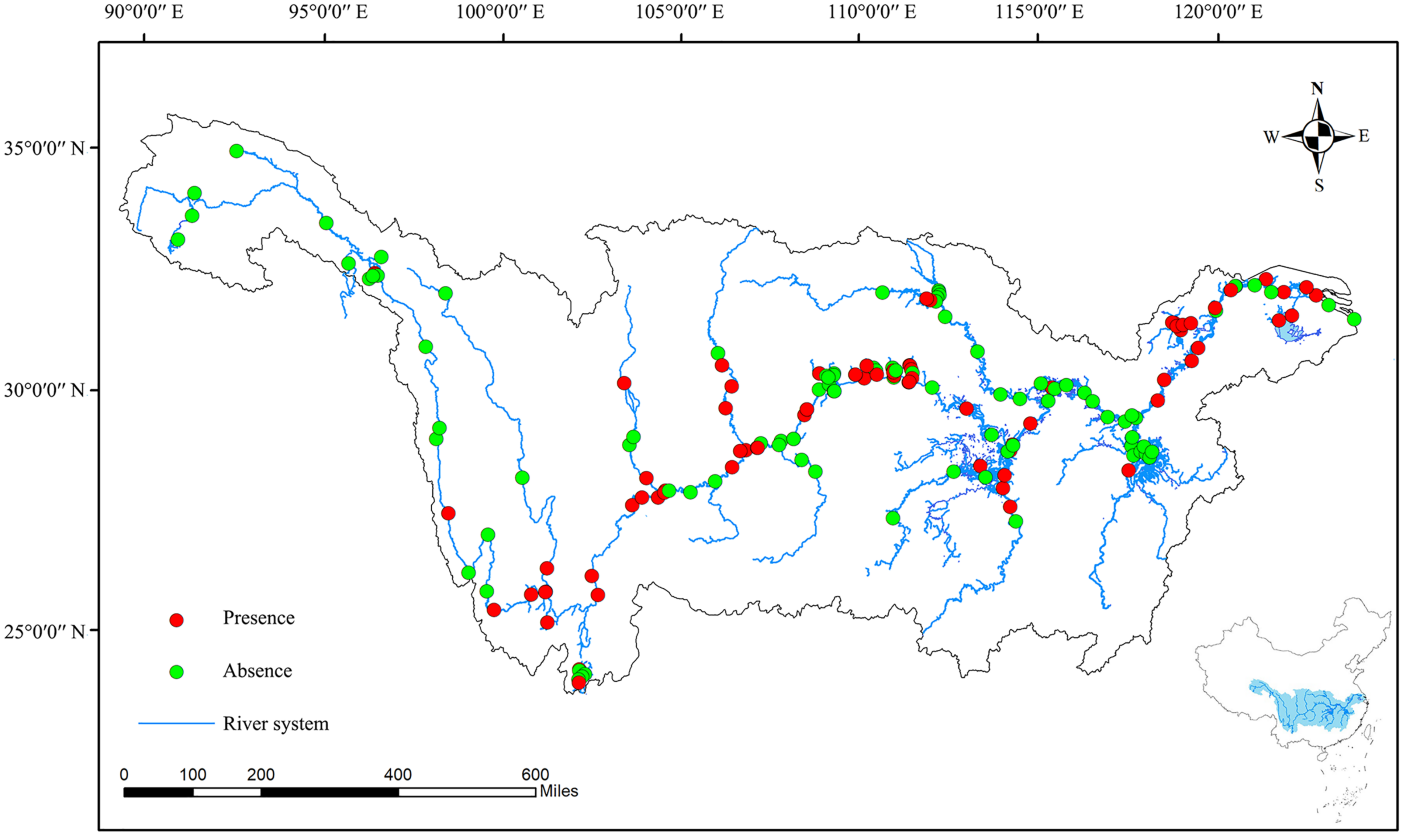
Presence and absence of colonial volvocine algae in each sampling sites.

Water samples were collected in the Yangtze River basin during summer (July to October) in 2020. A total of 172 sampling sites were set from the source region to the estuary area of the Yangtze river basin, the sampling area included the mainstream of the Yangtze river (the source region, the Jinsha river, the upper, middle, lower, and estuary region), the primary tributary of the Yangtze river (the Yalong Jiang River, the Minjiang River, the Wujiang River, the Jialin Jiang River, the Xiangjiang River, the Yuanjiang River, the Hanjiang River, and the Ganjiang River), and the reservoirs and lakes (the Dianchi Lake, the Dongtinghu Lake, the Poyanghu Lake, the Chaohu Lake, the Taihu Lake, the Danjiangkou Reservoir, and the Three Gorges Reservoir) (Figure 1).

For each sampling site, 1.5 l of water was collected to a sterile PET bottle and immediately transported to an adjacent laboratory at a low temperature of 0 ~ 4°C. Following that, all the water was filtered through 0.22 μm polycarbonate membranes (Millipore, United States), which were then kept frozen at −80°C until DNA extraction.

The longitude, latitude, and elevation of each sampling site were recorded using a handheld GPS device (Magellan, United States) in the field. Water physicochemical parameters, including total phosphorus (TP), total nitrogen (TN), ammonium nitrogen (NH_4_^+^-N), COD, BOD, Chlorophyll *a* (chl*a*), and COD_Mn_, were measured according to the Water and Waste Water Monitoring and Analysis Standard Methods (Editorial Committee of Ministry Environmental protection of China, 2002). The other conventional water parameters, including dissolved oxygen (DO), water temperature (Temp), pH, and conductivity (SPC), were measured by a portable multi-parameter water quality analyzer (Xylem) *in situ*.

### 2.2. DNA extraction, PCR amplification, and sequencing

Genomic DNA was extracted using the DNA extraction Kit (TGuide S96 Magnetic Universal DNA Kit, TIANGEN) according to the manufacturer’s protocols. Then, the full-length 18S ribosomal RNA gene was amplified by PCR (95°C for 4 min, followed by 35 cycles at 98°C for 15 s, 55°C for 30 s, and 72°C for 2 min, and a final extension at 72°C for 7 min) using barcoded primers Euk-A (AACCTGGTTGA TCCTGCCAGT) and Euk-B (GATCCTTCTGCAGGTTCACCTAC) (Full-length, ∼2,000–2,540 bp) (Countway et al., 2005; Callahan et al., 2019; Liu et al., 2021). PCR reactions were performed in triplicate 30 μL mixture containing 11.7 μL of nuclease-free water, 15 μL of PCR Mix (KOD OneTM PCR Master Mix, Toyobo), 1.8 μL of each primer (5 μM), and 1.5 μL of template DNA.

Amplicons were extracted from 2% agarose gels and purified using the Monarch DNA Gel Extraction Kit (New England Biolabs, Ipswich MA, United States) according to the manufacturer’s instructions and quantified using a microplate reader (GeneCompang Limited, synergy HTX). Purified amplicons were pooled in equimolar amounts and sequenced on the PacBio Sequel II platform (PacBio, United States).

### 2.3. Bioinformatics analysis

Raw reads were demultiplexed using lima v1.7.0 (github.com/pacificbiosciences/barcoding) based on barcode sequence, then the circular consensus sequence (CCS) reads were generated from the raw PacBio sequencing data by SMRT link v8 (www.pacb.com/products-and-services/analytical-software/smrt-analysis/). The adapter sequences in CCS were removed by Cutadapt v1.17 (Martin, 2011). Then, the CCS were analyzed using the software package DADA2 in R (Callahan et al., 2016), and the parameters were set according to the study of reference (Callahan et al., 2019).

Although Volvocales also contains other colonial flagellate algae, such as Spondylomoraceae and Haematococcaceae, these species are uncommon and with low concern, hence they are not included in the scope of this study (Desnitskiy, 2020), so we only consider algae belong to Volvocaceae, Goniaceae, and Tetrabaenaceae. For the taxonomy assignment, we built the reference database of CVA according to the study of Wang et al. (2019). All colonial volvocine algae sequences of 18S rRNA reads were downloaded from the NCBI Nucleotide database,^1^ short reads (less than 1,500 bp), and redundant reads were eliminated by CD-HIT (Fu et al., 2012), then the phylogenetic analysis was performed by MAFFT v7.490 (Katoh and Standley, 2013) and FastTree v2.1.11 (Price et al., 2010), and incorrect reads were discarded. The taxonomical assignment of each amplicon sequence variants (ASVs) was analyzed by BLASTn search with BLAST+ (Camacho et al., 2009) against the local reference database. Each ASV that had been annotated as CVA had its sequence further blasted to the NT database using a BLAST online search.^2^ The taxonomic information of each ASV was manually checked, and if the results did not match with each other, the ASVs were discarded. Finally, ASVs that were blasted to the local reference with a similarity greater than 98.8% were then used for further analysis.

### 2.4. Statistical analysis

The categorical variable data do not necessarily have a linear relationship with the scaled data, so the Eta-squared value was used as the correlation coefficient to determine the correlation between the existence of CVA and environmental factors, the Eta-squared values of 0.14, 0.06, and 0.01 were considered a strong, moderate, and weak correlation, respectively (Toloraia et al., 2022). The Eta-squared function in the “Lsr v0.5.2” R packages was used to calculate the Eta-squared value.

The Random Forest Algorithm was also utilized to determine the relationship between the presence of CVA and environmental parameters (Altitude, Temp, pH, SPC, BOD, DO, COD_Mn_, NH_4_-N^+^, COD, TN, TP). The “randomForest v4.7.1.1” R packages were used to implement the Random Forest algorithm. There are 172 dataset groupings used in total. For creating a random forest model, 70% of dataset groups are randomly chosen as training samples, while 30% of dataset groups are randomly chosen as test samples. Inappropriate values of ntree and mtry may lead to the model being underfitted or overfitted. An increased for-loop was used to determine the best parameter value, and the mean square error was used to assess the model’s correctness. Finally, the best parameters of our model are mtry = 2 and ntree = 400.

The Canonical correspondence analysis (CCA), Detrended correspondence analysis (DCA), Variance Inflation Factor (VIF), and Mantel test were performed to analyze the association between the CVA community composition and environmental conditions using the R “vegan v2.6.4” package. For community species data with lots of zero values, Hellinger’s transformation was applied (Legendre and Gallagher, 2001), environmental factors with VIF of more than 10 were eliminated (Kennedy, 1992), and DCA was then used to analyze the data, CCA was utilized for analysis because it was discovered that the maximum axis length was greater than 4 (Li and Kendrick, 1995).

To reveal how the pollution gradient variation affects CVA distribution along the basin, 10 environmental parameters (TP, TN, NH_4_^+^-N, COD, chl*a*, COD_Mn_, DO, Temp, pH, and SPC) were selected and normalized by Z-score normalization, then the average value of all environmental parameters was used as the stress index, and all samples were split into low pollution and high pollution using the 50th percentile of the stress index as boundaries (Rooney and Bayley, 2010). For these two groups, the co-occurrence network was explored based on the Spearman’s correlation matrix assembled with R package “igraph v1.3.5” (Csardi and Nepusz, 2006) and visualized based on Gephi v0.9.1 software according to related study (Zhang et al., 2020).

MaxEnt software 3.4.1 (Princeton University, Princeton, NJ, United States) was used to analyze the potential distribution of CVA. The occurrence data of CVA were obtained from this study. The WorldClim v2.1^3^ provided the environmental variables we used for this study, the standard 19 WorldClim Bioclimatic variables (Details of each bioclimatic variable please refer to^4^) of 1970–2000 were download as the near current data, the SSP1-2.6, SSP2-4.5, SSP3-7.0, and SSP5-8.54 emission scenarios of 2021–2040 were download as the future data, and the GCM chose the BCC-CSM2-MR. The obtained species distribution data and the current climate factor data were imported into MaxEnt v3.4.4 software. The environment factor was set as a continuous variable, a response curve was created, and the jackknife method was used. 25% of the data were chosen as the test set, 75% as the training set, and other parameters were set to default values. We repeated the analysis ten times. The contribution rates of 19 climate factors were simulated by the jackknife method. Factors with contribution rates greater than 0 were retained. Pearson correlation coefficients were analyzed for the retained climate factors. All the factors were kept if the correlation coefficients were less than 0.8. If the correlation coefficient is greater than 0.8, compare the contribution rate from the original simulation and keep the climate factors with a higher contribution rate. Finally, seven climatic components were chosen to reconstruct the maximum entropy model of CVA in the Yangtze River basin, including Isothermality (Bio3), Temperature Annual Range (Bio7), Mean Temperature of Wettest Quarter (Bio8), Mean Temperature of Warmest Quarter (Bio10), Mean Temperature of Coldest Quarter (Bio11), Precipitation of Wettest Quarter (Bio16), and Precipitation of Coldest Quarter (Bio19).

## 3. Results

### 3.1. Biogeography patterns of colonial volvocine algae communities

In this work, we used the PacBio Sequel II high-throughput sequencing method to analyze 172 samples. For these samples, the raw CCS ranged from 5,000 to 7,629, with its average length ranging from 1,690 to 1825 bp. After proceeding by DADA2, a total of 9,309 ASVs were retrieved from this method, and the sequence length ranged from 1,225 bp to 2005 bp, with an average value of 1771 bp, a median value of 1753 bp (Supplementary Figure 1). After annotating with the local database, 34 ASVs belonging to CVA were classified. For the CVA sequences, the sequence length ranged from 1740 bp to 1747 bp, with an average value of 1743 bp, a median value of 1743 bp.

A total of 172 sampling sites were set in this study, CVA was discovered in 76 sampling sites, which are distributed in the upper, middle, and lower areas of the Yangtze River, various lakes/reservoirs, and some tributaries (Figure 1; Supplementary Figure 2). For the mainstream of the Yangtze River, CVA is present in the upper, middle, and lower areas of the Yangtze River, but not in the source area of the Yangtze River (Tibet and Qinghai Province). For the primary tributaries, only five of the eight tributaries have found CVA (including Yalong River, Minjiang River, Jialing River, Hanjiang River, and Xiangjiang River). For lakes/reservoirs, all of the lakes and reservoirs in the Yangtze River basin have found CVA (the Dianchi Lake, the Dongtinghu Lake, the Poyanghu Lake, the Chaohu Lake, the Taihu Lake, the Danjiangkou Reservoir, and the Three Gorges Reservoir).

**Figure 2:**
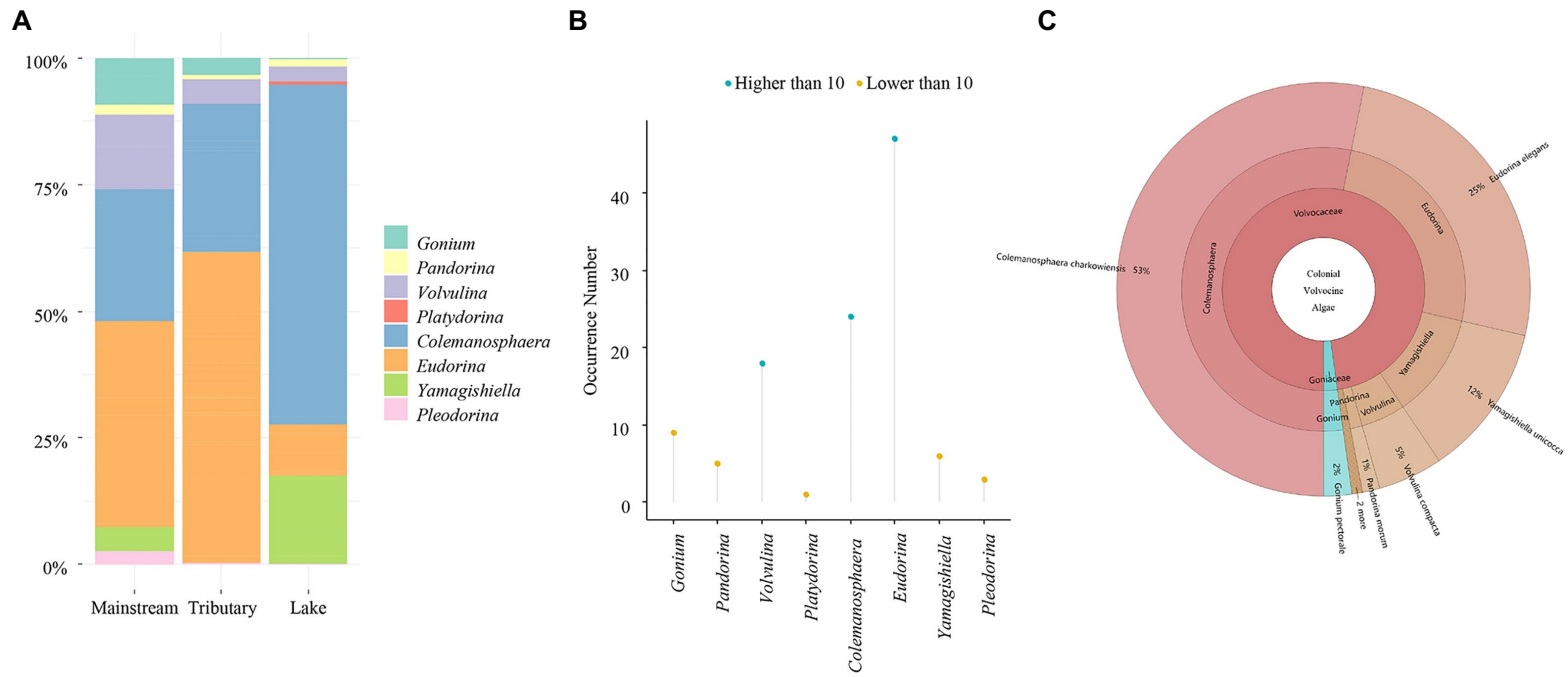
Relative abundance and occurrence number of colonial volvocine algae in the Yangtze River Basin. **(A)** Relative abundance. **(B)** Occurrence number. **(C)** Relative abundance of each species.

*Gonium*, a member of the Goniaceae family, and *Pandorina*, *Volvulina*, *Platydorina*, *Colemanosphaera*, *Yamagishiella*, *Eudorina*, and *Pleodorina*, members of the Volvocaceae family were among the 2 families and 8 genera discovered in this study. Except for *Platydorina*, most of the genera are widely dispersed around the globe. The community composition of CVA is depicted in Figure 2A. The Yangtze River’s mainstream and its tributaries share a similar community structure, with *Eudorina* and *Colemanosphaera* being the dominant species. *Colemanosphaera* and *Yamagishiella* are the two dominate genera in lake, the relative abundance of other genera is minimal. *Eudorina*, *Colemanosphaera*, and *Volvulina* are the three genera discovered in the Yangtze River basin with a frequency higher than 10, and all other genera have a frequency of finding below ten (Figure 2B). For the species level, 9 species were discovered in our study, including *Colemanosphaera charkowiensis*, *Eudorina elegans*, *Gonium pectorale*, *Pandorina morum*, *Platydorina caudata*, *Pleodorina illinoisensis*, *Pleodorina starrii*, *Volvulina compacta,* and *Yamagishiella unicocca*. The top two species with the highest relative abundance were *Colemanosphaera charkowiensis* and *Eudorina elegans* (Figure 2C). In conclusion, *Eudorina* and *Colemanosphaera* are the main dominant group in CVA in the Yangtze River basin, with a high relative abundance and extensive distribution (high frequency of detection).

### 3.2. Environmental effects on the presence/absence of colonial volvocine algae

**Figure 3:**
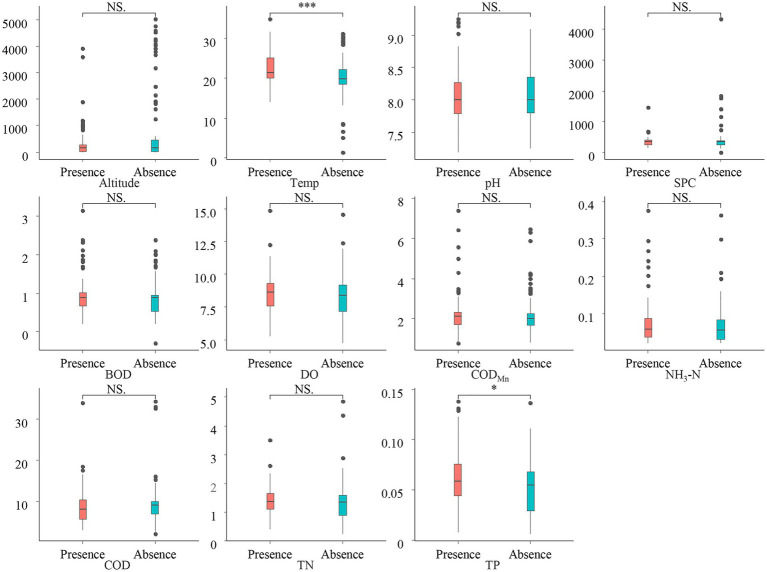
Environmental difference between presence/absence sites of colonial volvocine algae. *p < 0.05; ***p < 0.001; NS, Not significant.

We conduct a Wilcoxon rank-sum test on the differences in environmental factors between CVA Presence/Absence sampling sites to examine the influence of environmental factors on the CVA biogeographical distribution. The results (Figure 3) showed that the Temp and TP at the CVA presence sites have a significantly higher value than those areas without CVA.

**Figure 4:**
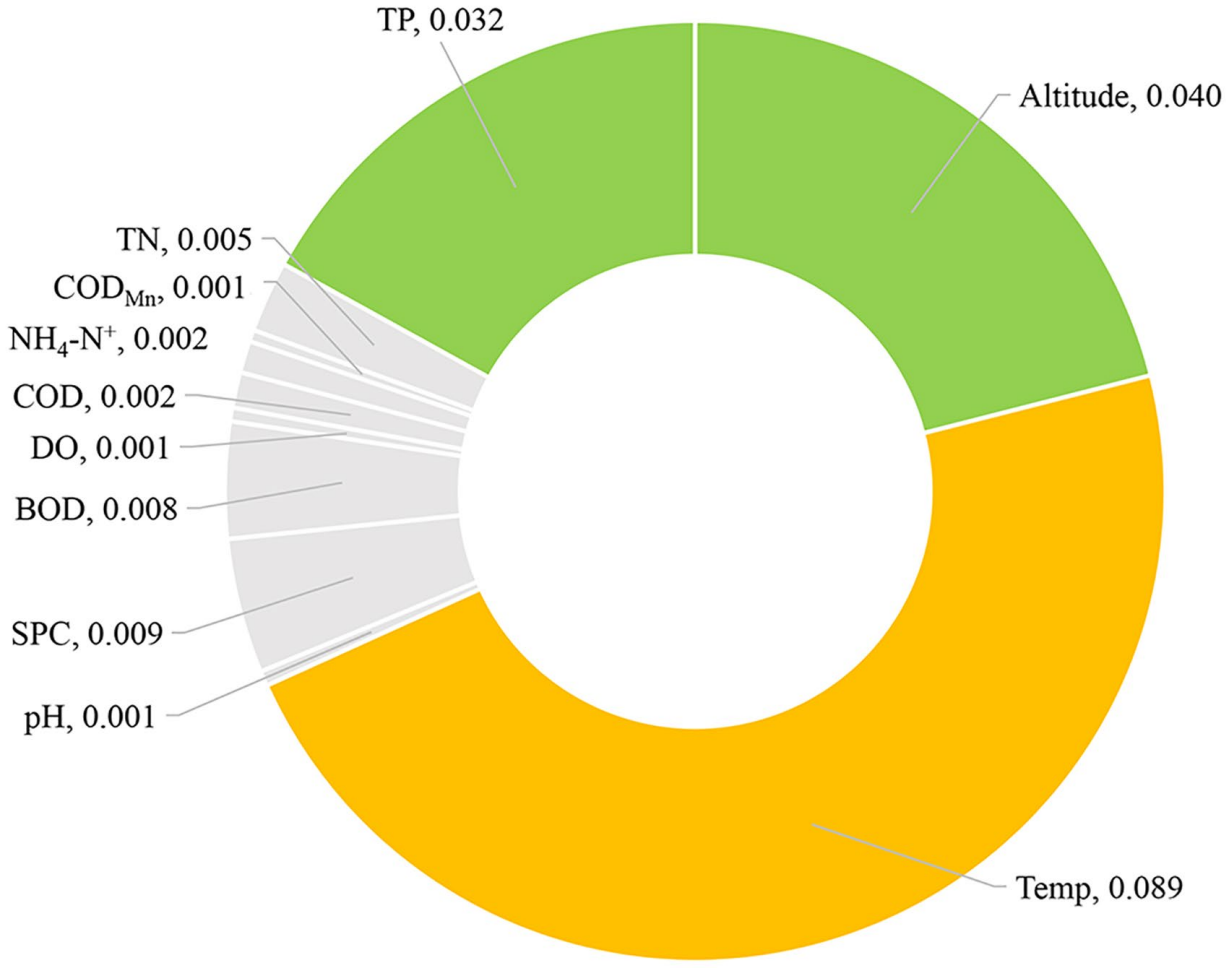
Eta-squared value of different environmental factors for the presence/absence of colonial volvocine algae.

**Figure 5:**
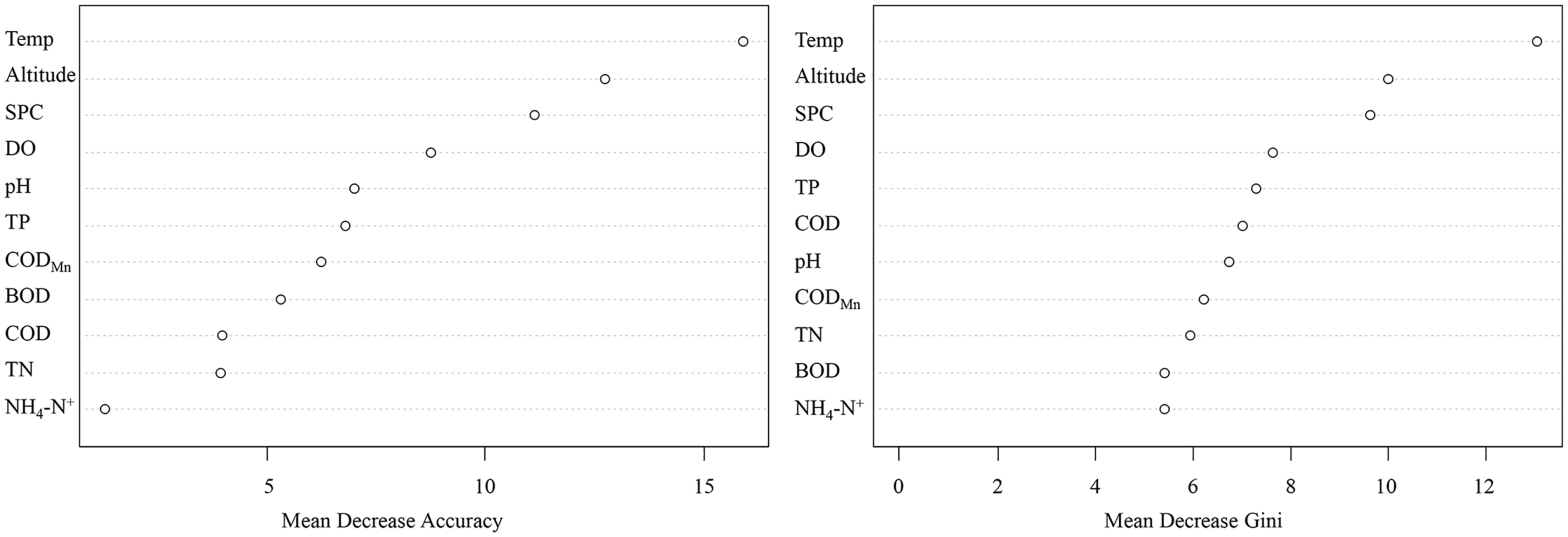
The importance of different environmental factors to the presence/absence of colonial volvocine algae based on random forest model.

To further evaluate whether environmental factors will have a major impact on the existence of CVA, we employed the Eta-squared value in statistics and the random forest method in machine learning. Temp, Altitude, and TP are environmental parameters with Eta-squared values greater than 0.01 (stronger than weak connection), as shown in Figure 4. The highest Eta-squared value for Temp among them is 0.089, which falls inside the model-strong correlation. Altitude and TP are in the weak-model association, with the Eta-squared values 0.04 and 0.032, respectively. Figure 5 displays the outcome of the random forest model. According to Calle and Urrea (2011), mean decrease accuracy (MDA) and mean decrease Gini (MDG) can both be used to quantify the importance of a variable. These two different measures of importance revealed the same conclusion: Temp, Altitude, and SPC have the greatest influence on the existence of CVA.

The results of two different analysis approaches, the random forest model in machine learning or the correlation coefficient employed in statistics, demonstrate that Temp and altitude are significant environmental factors determining the occurrence of CVA. Further evidence shows that TP is a significant environmental factor influencing the existence of CVA comes from the difference in environmental factors between CVA Presence/Absence sample sites and Eta-squared value.

### 3.3. Environmental effects on community structure of colonial volvocine algae

**Figure 6:**
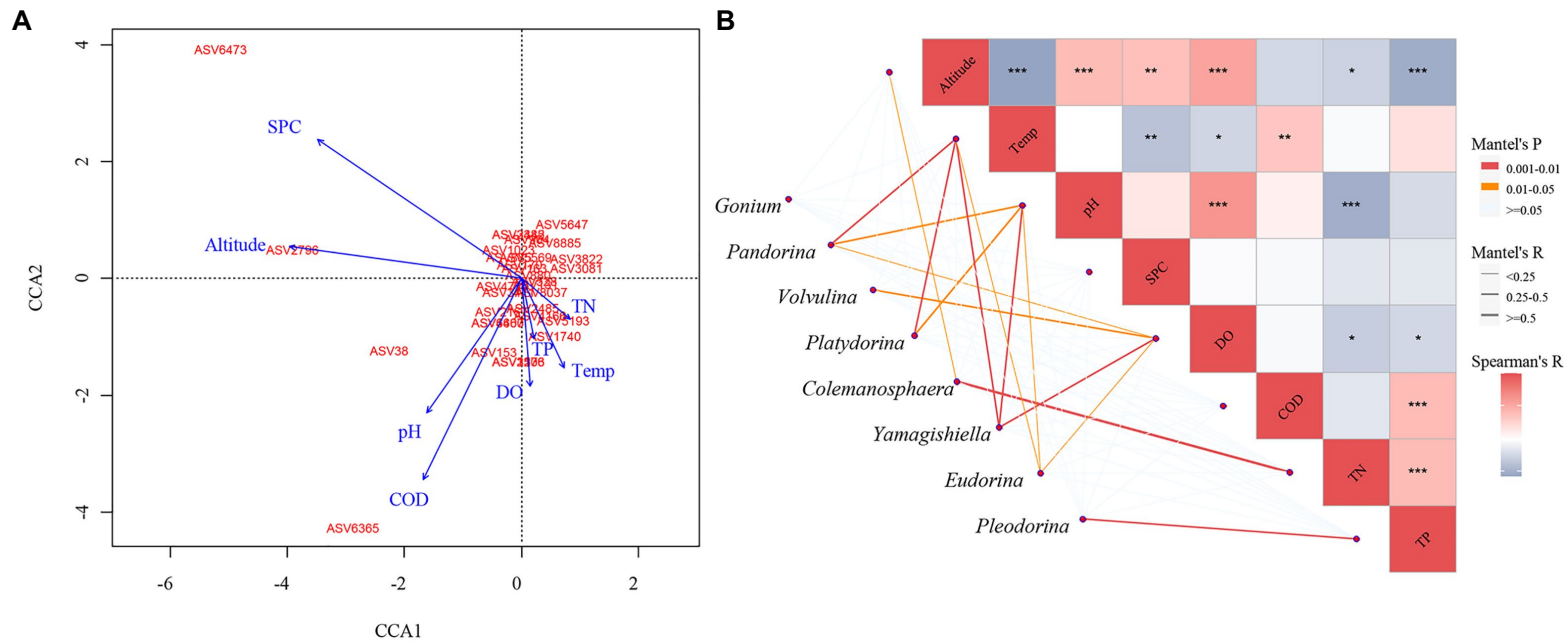
The CCA analysis and Mantel test between the different environmental factors and colonial volvocine algae community. **(A)** CCA analysis. **(B)** Mantel test.

We further analyzed which environmental factors will have a substantial impact on the community composition of CVA. The community structure of CVA for the entire community as determined by CCA (Figure 6A) is influenced by SPC, Altitude, pH, COD, Temperature, and DO (*p* < 0.05). Utilizing the Monte Carlo fitting method, it was shown that SPC (*R*^2^ = 0.69, *p* < 0.01) and Altitude (*R*^2^ = 0.67, *p* < 0.01) are the variables that have the most explanatory power for the CVA community.

We further analyzed the influence of each environmental factor in each genus (Figure 6B). The environmental factors significantly affected each genus are different, with R^2^ > 0.25 and p < 0.05 as the standards: the community structure of *Pandorina* and *Platydorina* is significantly affected by Temp and pH. *Volvulina* is significantly affected by DO. *Colemanosphaera* is significantly affected by TN. *Pleodorina* is significantly affected by TP. *Yamagishiella* is significantly affected by Temp, pH, and DO.

### 3.4. Impacts of anthropogenic pollution on CVA interspecific co-occurrence pattern

Rivers are under multiple threats from land-use change, fragmentation, and pollution (Li et al., 2020), and it has been estimated that anthropogenic sources are predicted to contribute the majority of river nutrients (Beusen et al., 2022). The algae community that makes up river ecosystems would suffer greatly from the extensive discharge of pollutants. Network co-occurrence patterns can show how ecosystems evolve in response to intervention (Woodward et al., 2010a,b). Research on biodiversity and ecosystem function has suggested using an ecological network as a conceptual framework (Thompson et al., 2012). So, ecological network analysis is a strong and potentially effective technique for monitoring and evaluating biological processes (Compson et al., 2018).

**Figure 7:**
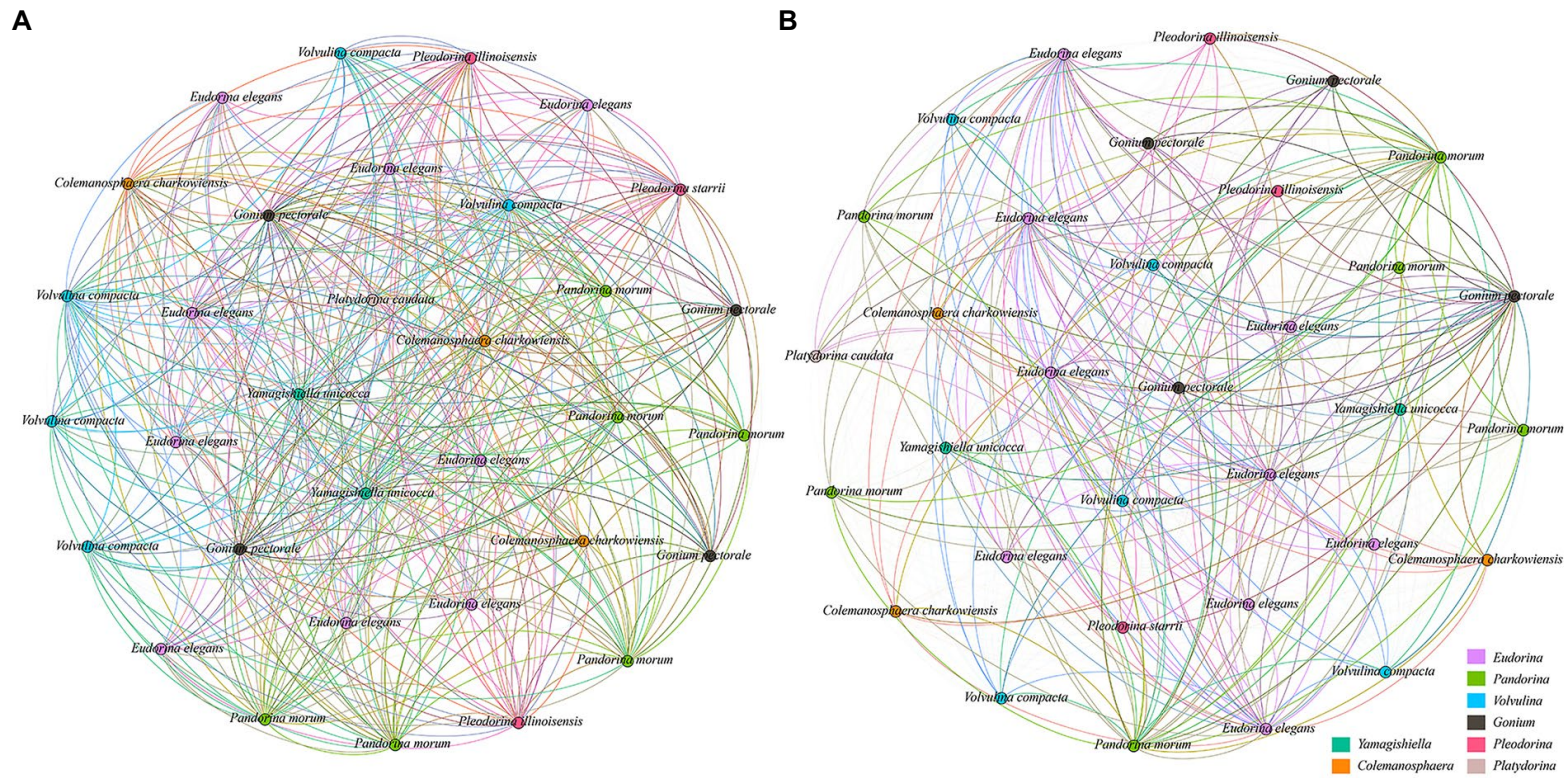
Ecological interaction network between CVA at each stress level based on the Spearman correlation analysis. **(A)** Low pollution. **(B)** High pollution.

Here, unique network topology was observed in co-occurrence patterns of different taxonomic groups at each stress level in the Yangtze River basin. The number of nodes was the same in low pollution (Figure 7A) and high pollution (Figure 7B) levels based on the network analysis, but the low pollution level has the higher edges number (528), average degree (32), graph diameter (0.06), graph density (1), and clustering coefficient (1) than in high pollution level, and the corresponding values of each feature in high pollution level were 522, 31.64, 0.05, 0.98, and 0.98, respectively. So, these features suggest a more complicated ecological interaction in the low pollution level.

### 3.5. Potential distribution of colonial volvocine algae in the Yangtze River basin

The suitability of the CVA geographical simulating distribution is evaluated using the ROC curve. The accuracy and dependability of the simulation results are very high, as shown by the ROC curve of the current climate conditions, where the AUC value reaches 0.924 (Ma et al., 2021). This information can be used to study the simulation of the potential distribution pattern of CVA.

**Figure 8:**
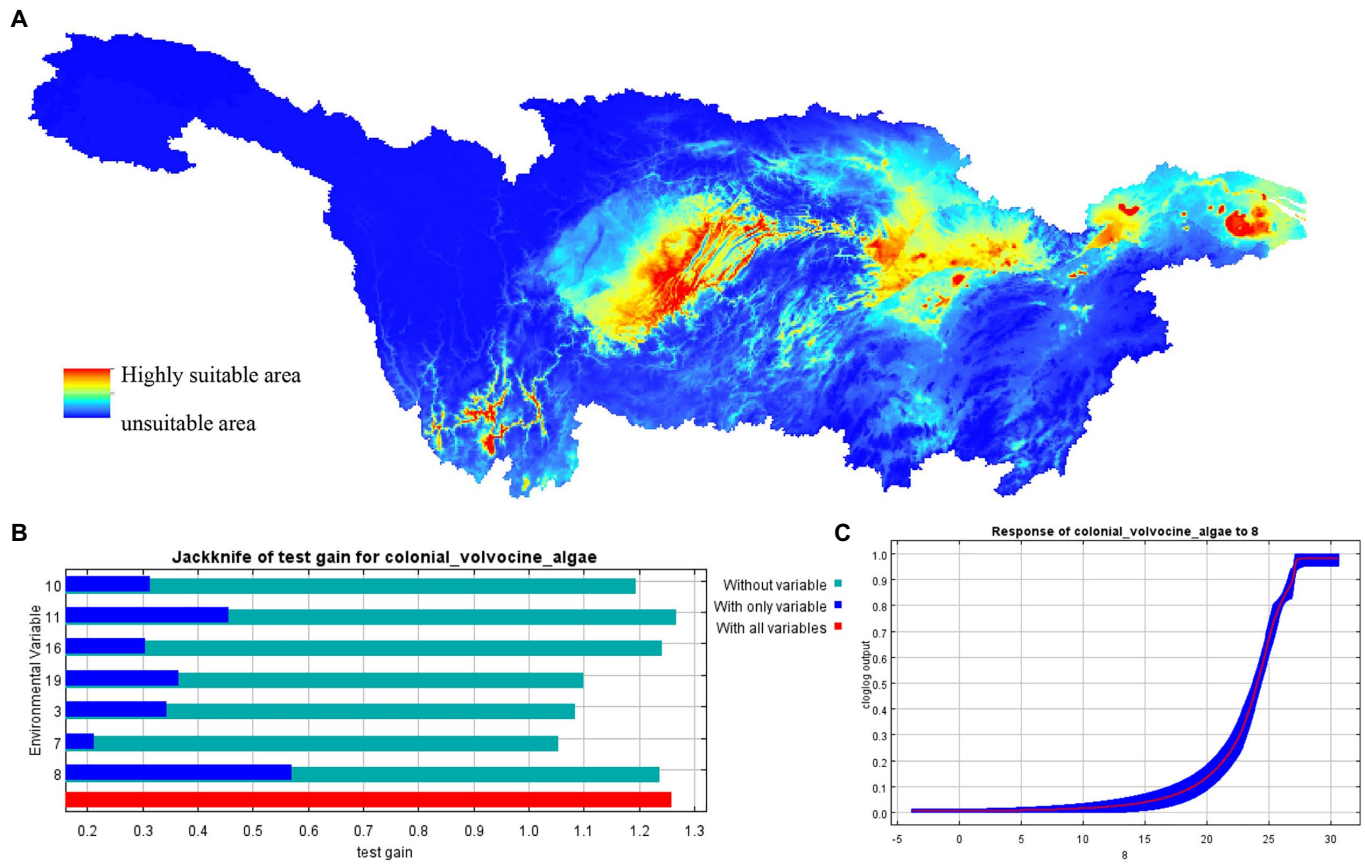
MaxEnt model predicted the distribution of colonial volvocine algae in current period. **(A)** Current distribution of CVA in the Yangtze River basin. **(B)** Jackknife analysis results showing most important environmental variables predicting potentially suitable distribution areas of CVA in the Yangtze River basin. **(C)** Response curves between the distribution probability of CVA and environmental variables (Bio8).

In the Yangtze River Basin, CVA is distributed between 24.71°~ 33.07° N and 97.17°~ 121.05° E, reaching Yunnan Province in the west and the Yangtze River Estuary in the east (Figure 8A). The main distribution areas of CVA include Yunnan province, the Three Gorges Reservoir Area, Hubei province, Anhui province, Jiangsu province, and Shanghai. The findings of the jackknife analysis reveal that among the examined variables, the Mean Temperature of West Quarter (Bio8) is dominant (Figure 8B). When the average temperature of the wettest quarter between 15 and 28°C is the most suitable temperature for CVA distribution, according to the relationship between Bio8 and the probability of CVA existence (Figure 8C).

**Figure 9:**
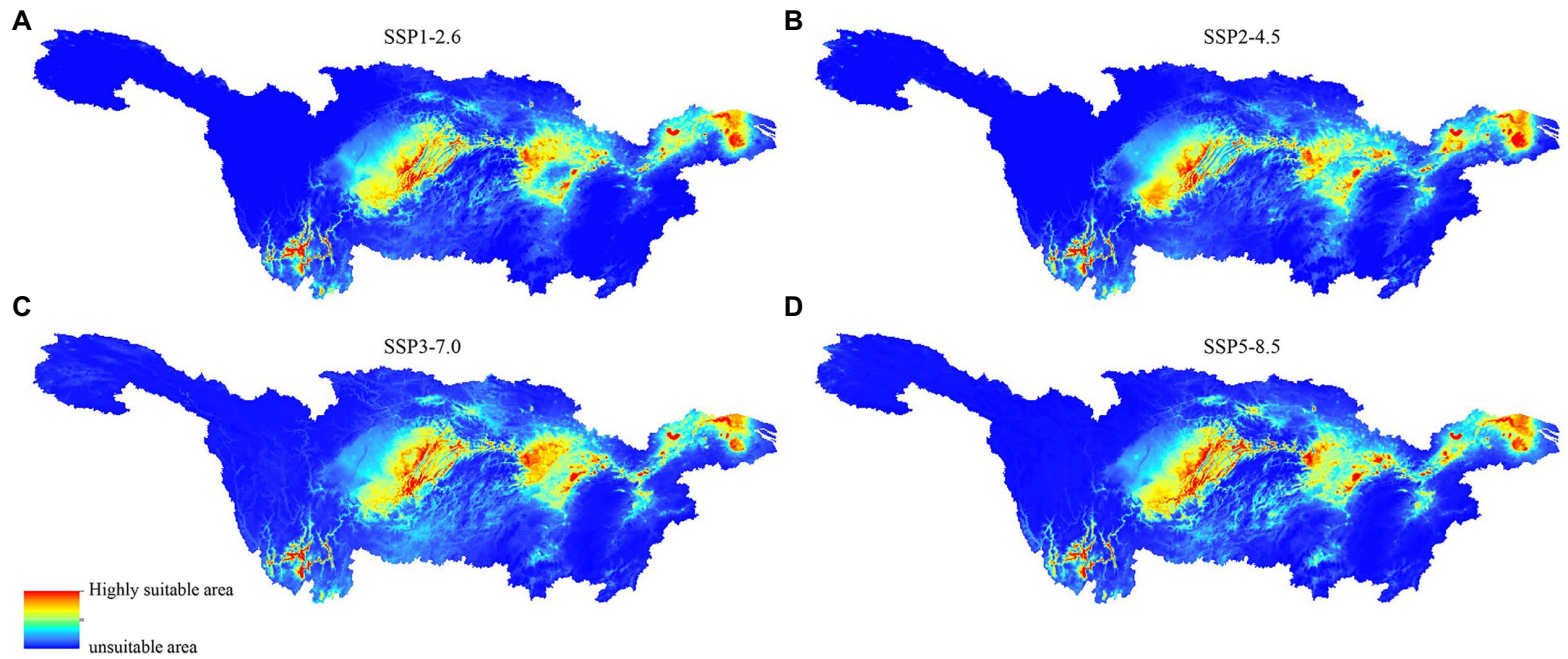
MaxEnt model predicted the distribution of colonial volvocine algae in future period under different climate scenarios. **(A)** SSP1-2.6. **(B)** SSP2-4.5. **(C)** SSP3-7.0. **(D)** SSP5-8.5.

From low-emission sustainable development to high-emission conventional development, the four emission scenarios SSP1-2.6, SSP2-4.5, SSP3-7.0, and SSP5-8.5 reflect the four most prevalent scenarios. Figure 9 illustrates how the distribution features of CVA-suitable locations vary significantly under the following four emission scenarios. Under the SSP1-2.6 scenario, the Three Gorges reservoir area contracts, which is primarily responsible for the decline in the high suitability area of CVA. Under the SSP2-4.5 scenario, the overall suitable area shrinks and is only slightly extended in the Taihu Lake region in Jiangsu Province. The distribution of highly suitable areas for CVA is becoming more fragmented under the two high-emission scenarios SSP3-7.0 and SSP5-8.5, and the unsuitable areas are much greater than the average level in the recent 30a. The Yangtze River’s mainstream, Taihu Lake, and other unsuitable places in Chongqing and Hubei, for instance, exhibit a growing trend. The four scenarios generally demonstrated a decline in the applicability of CVA.

## 4. Discussion

### 4.1. Biogeography patterns of colonial volvocine algae communities

The CVA found in this study is only present in limited rivers, but distributed throughout the basin in all lake/reservoir areas. The lake/reservoir environment is more likely ideal for the distribution of CVA species since it has a lower flow rate than rivers, which has a substantial impact on phytoplankton’s growth and reproduction (Marshall and Burchardt, 1998).

*Eudorina* and *Colemanosphaera* are the two most dominant groupings of CVA in the Yangtze River basin. *Colemanosphaera* is a genus with similar shape to *Eudorina* (Nozaki et al., 2014). According to a related study (Wehr et al., 2015), *Eudorina* is the most frequently seen and encountered species among green algae. CVA includes a wide range of morphologically simple to complicated categories, like *Volvox* and *Pleodorina* have more complex morphologies than *Eudorina*. They have evolved different forms and functions, which represent transitions in the units of fitness (Herron and Michod, 2008). This should make these species more adaptable, but their distribution range and abundance are smaller in this study, the database may be the cause. In the local database we created for this study, there are 658 *Eudorina* sequences and 342 *Pleodorina* sequences. Since *Eudorina* has more reference sequences than *Pleodorina* does, more of its ASVs are annotated, whereas some *Pleodorina* ASVs might not be.

The scarce CVA species *Platydorina* was also discovered in our investigation. This species was last reported in China in 2009 when it was discovered in the Shenzhen Reservoir (Hong et al., 2010). However, Coleman did not discover it 2 year later (Coleman, 2014). Since then, there are not many reports of *Platydorina* in China or even globally. In this study, *Platydorina* was discovered in Chaohu Lake at a single sampling location (M158 site). This is another possible suitable distribution area for *Platydorina* in China, which offers a specific area for *Platydorina* sample collection.

### 4.2. Environmental effects on the presence/absence of colonial volvocine algae

According to the result of environmental factors’ difference, Eta-squared number and the Random Forest model, the main variables determining the occurrence of CVA may be Temp, Altitude, and TP. Water temperature is a key factor affecting the diversity and abundance of phytoplankton. Different water temperatures will affect the nutrient absorption efficiency, enzyme activity, cell metabolism, and growth of phytoplankton. For most algae, the rise of water temperature within the appropriate temperature range is conducive to the growth and reproduction of phytoplankton (Yang et al., 2019). This study found that the water temperature at sampling sites where CVA exists is 14.1 ~ 34.8°C, and the average water temperature is 22.88°C, which is within the temperature range of CVA cultivation (Hu et al., 2019, 2020). This water temperature may be a suitable temperature range for the distribution of CVA.

The growth of phytoplankton depends on the nutritional element phosphorus. It takes part in several crucial metabolic activities in cells, such as catabolism, energy transformation, cell tissue coordination, and cell structure (Cembella et al., 1982). Phytoplankton in rivers can directly consume or assimilate numerous phosphorus compounds in different forms to receive phosphorus sources for growth. Generally, a body of water is regarded to be restricted for the growth of phytoplankton if the concentration of dissolved active phosphorus is less than 0.01 mg/L (Reynolds, 1984). According to numerous researches, phosphorus limitation occurs in the summer (Komarkova and Hejzlar, 1996). This is a result of the high summer temperatures and the quick phytoplankton growth rate. The above argument is supported by the study’s finding that TP and CVA have a substantial association. TP concentrations between 0.01 and 0.14 mg/L, with an average of 0.06 mg/L, are appropriate for the growth of CVA, according to our environmental factors data.

Altitude plays a crucial role in the presence of CVA. Our research revealed that the ideal height for CVA is 0–3,920 m and that CVA cannot exist over 3,920 m. Altitude is not technically an environmental influence. Pressure, temperature, precipitation, and other environmental elements in water bodies will alter dramatically as altitude changes, and there may be distinct social and economic conditions, as well as differing levels of natural disturbance (Han et al., 2021). The change in these variables will have an impact on phytoplankton growth and distribution (Li et al., 2021). Altitude influences the distribution of CVA in the Yangtze River basin by affecting various environmental factors conditions, and it also plays a significant role in indirectly regulating the biographical distribution of CVA, as demonstrated by the results of this study, which show that altitude is significantly related to Temp, pH, SPC, DO, TN, and TP (Figure 6B).

### 4.3. Environmental effects on community structure of colonial volvocine algae

The relation between environmental factors and CVA community composition was further investigated in this study. According to the CCA and mantel test result, *Pandorina*, *Platydorina*, and *Yamagishiella* are all highly impacted by Temp, while *Pleodorina* is primarily impacted by TP. These two environmental factors have been discussed in the section above.

The composition of *Pandorina*, *Platydorina*, and *Yamagishiella* is greatly impacted by pH. Phytoplankton’s distribution and growth are impacted by pH due to how it affects their photosynthesis (Chen and Durbin, 1994). For instance, it is advantageous for algae to absorb CO_2_ for photosynthesis in weakly alkaline water bodies. pH also directly affects the growth and reproduction rate of algae (Agrawal, 2012). Meanwhile, pH can affect the form, proportion, and transformation of nutrient elements (nitrogen and phosphorus) in water (Körner et al., 2001; Zhao et al., 2022), thus indirectly affecting the distribution and succession of CVA.

DO can have a significant impact on *Volvulina* and *Yamagishiella*. Without dissolved oxygen, phytoplankton cannot grow and reproduce. Through photosynthesis, phytoplankton can release oxygen, increasing the dissolved oxygen level in the water body. Phytoplankton respiration will consume the water body’s dissolved oxygen (Smith and Piedrahita, 1988). In addition, as organic matter degrades following phytoplankton death, it will absorb dissolved oxygen in the water body (Boyd et al., 1975). There are numerous chemistry-based reasons to assume a causal relationship between water temperature and dissolved oxygen (Post et al., 2018), and the correlation between environmental factors demonstrates that DO is also significantly correlated with various environmental factors (Figure 6B). DO and pH are positively associated. An increase in dissolved oxygen will result in a decrease in the concentration of H^+^(O_2_ + 4H^+^+4e^−^ = 2H_2_O), thus raising the pH (Li et al., 2015). The phytoplankton will release oxygen while absorbing CO_2_ through photosynthesis, causing a significant buildup of bicarbonate in the water body and raising pH (Wurts and Durborow, 1992). In addition, the strong relationship between DO and TN, TP also controls the composition of the phytoplankton community by controlling the concentration of nutrients (Khalid et al., 1978).

TN has a huge impact on the *Colemanosphaera* community’s composition. One of the crucial nutrients for the growth and reproduction of algae is TN. The organization of the algae community may vary because of TN concentration changes (Glibert et al., 2016). According to certain research, the growth of algae will be restricted when TN concentrations are less than 1 mg/L (Zhu et al., 2016), and even in eutrophic lakes, the TN threshold of indicator species is only approximately 1.6 mg/L (Cao et al., 2016). In this study, we discovered that TN concentrations at sampling sites with *Colemanosphaera* presence ranged from 0.41 ~ 3.50 mg/L. Among the 21 sampling sites with the presence of *Colemanosphaera*, six sampling locations had TN concentrations greater than 1.6 mg/L and six had TN concentrations less than 1 mg/L. The fact that nearly half of the sites are above the TN appropriate level suggests that *Colemanosphaera* can withstand a more extreme TN concentration.

### 4.4. Impacts of anthropogenic pollution on CVA interspecific co-occurrence pattern

The low pollution group has higher “edges number,” “average degree,” “nodes connectivity,” “edges connectivity,” “graph diameter,” “graph density,” and “clustering coefficient.” Among them, “edges number” is the number of edges in the network, and “average degree” is the average number of connections of all nodes in the network, a larger number of these two parameters indicated a larger network size (Layeghifard et al., 2017). “Graph diameter” is the maximum distance between all nodes, “graph density” is the density of the network, and a larger number of these two parameters indicated a closer relationship between network nodes (Luke, 2015). “Clustering coefficient” is used to reflect the compactness between an adjacent node in a network, the higher value indicated a higher aggregation degree of species, so the network has higher connectivity (Watts and Strogatz, 1998). The above indicators reflect the scale and complexity of the network. The larger the value is, the larger the scale, complexity, and high connectivity of the network are.

In our study, with the increase in pollution level, the high pollution group has reduced all the above indicators to a certain extent. Some studies have pointed out that the increase in pollution level will affect various indicators of the network, such as the reduction of network complexity caused by pollution (Zappelini et al., 2015), affects network connectivity and network density (Lupatini et al., 2014), and showed significant negative influence on the complexity and dependency of a network (Al et al., 2021). Therefore, with the enhancement of human activities and the interference of the environment, the disturbance of environmental factors will affect the entire CVA ecological network. The instability of the network structure increases, and the scale and connectivity decrease, leading to the reduction of the complexity of the CVA co-occurrence network and its diversity.

### 4.5. Potential distribution of colonial volvocine algae in the Yangtze River basin

Results from niche model simulations are most reliable when three factors are considered. First, when choosing a model, consider that various models’ prediction accuracy varies widely and that the MaxEnt model has greater prediction dependability than other models (Merow et al., 2013; Rong et al., 2019). The extent of sample collection and the uniformity of sample coverage come in second, the prediction accuracy increases with sampling point selection close to the true distribution of the species, and the data utilized in this study are representative of the true distribution of the species. The third consideration is the origin, nature, and precision of environmental elements. The prediction accuracy increases with the number of types and quantities of factors selected during simulation. To guarantee the types, amounts, and accuracy of data, the environmental factor data from this study were downloaded to the WorldClim database (Fick and Hijmans, 2017). The adequacy of the simulated geographical distribution is evaluated using the ROC curve. The AUC value exceeds 0.9 with a value of 0.924, suggesting strong prediction accuracy and trustworthy simulation findings (Ma et al., 2021).

The Three Gorges Reservoir Area, Chaohu Lake, and Taihu Lake are shown to be highly suitable growth locations for CVA. There is a high likelihood that CVA can be identified in these regions, even under different emission scenarios in the future. According to the environmental factors data, Chaohu Lake’s water temperature is 29–34°C, its altitude is 7–8 m, and its TP is 0.06–0.12 mg/L. The water temperature in Taihu Lake is close to 23°C, its altitude is 0 m, and TP is 0.06–0.1 mg/L. The water temperature in the Three Gorges is 14–20°C, its altitude is 135–457 m, and TP is 0.03–0.12 mg/L. The average water temperature, altitude, and TP concentration across all sites in the three regions are 22.78°C, 153 m, and 0.07 mg/L. These values are close to the average value of CVA existing sites. The three regions’ water types are all lake/reservoir kinds, which is congruent with the study’s findings.

The importance of the Mean Temperature of West Quarter was ranked first throughout all 10 iterations of MaxEnt analyses for this study. The other two factors that scored in the top three in importance were Bio3 (Isothermality) and Bio10 (Mean Temperature of Warm Quarter). The fact that these three environmental parameters are all connected to temperature suggests that it is a significant climate variable that limits the distribution of CVA. This conclusion is also compatible with the microscopic results because air temperature and water temperature are two closely associated environmental parameters (Toffolon and Piccolroaz, 2015). So, the temperature is a significant environmental element influencing the existence, distribution, and change in the community structure of CVA.

Under the four future emission scenarios, MaxEnt predicts that the potential distribution area of CVA may reduce, and the habitat of CVA may be fragmented, especially under the two high-emission scenarios of SSP3-7.0 and SSP5-8.5. Habitat fragmentation will result in the loss of the living area of CVA. Habitat fragmentation leads to fewer and fewer large habitats, while more and more small isolated habitats (Rodrıguez and Delibes, 2003), thus increasing the probability of extinction of rare species in CVA. Fragmentation will isolate habitats from each other and change population diffusion and migration patterns, population genetics, and variation, thus affecting species reproduction and migration capacity (Dos et al., 2007). Therefore, our research showed that CVA distribution may be seriously threatened by climate change. Meanwhile, we sampled the surface waters in an effort to reflect the CVA’s recent biodiversity (Deiner et al., 2017), as recent biodiversity can more accurately depict the interaction between the CVA community and environmental conditions. However, most CVA species are only found for a short time when they grow and reproduce in water, and the zygotes produced by CVA sexual reproduction may remain dormant in the mud/sediment for years or even decades before conditions are optimal for growth (Kirk, 1998; Umen, 2020). Since we only sampled the surface water onetime, more thorough temporal surveys and sampling of the sediment or mud would further consummate the potential distribution of CVA, thus offering more information to study the CVA habitat fragmentation.

## 5. Conclusion

In this study, the biogeography of CVA in the Yangtze River basin was investigated using metabarcoding technology, which is based on environmental DNA. CVA was discovered in the Yangtze River’s upper, middle, and lower areas, five major tributaries, and all lake and reservoir areas. Lakes/reservoirs are better suited for the distribution of CVA than rivers. Our investigation discovered 2 families, 8 genera, and 9 species. *Eudorina* and *Colemanosphaera* are the two main dominant groups. We also discovered several rare *Platydorina* species, pointing us on the right path for future taxonomic studies regarding sample collection.

We discovered that temperature, altitude, and TP are significant environmental factors that influence the distribution of CVA based on the differences in environmental factors, Eta-squared value, and random forest model. Among these, 14.1–34.8°C is the most ideal water temperature for CVA distribution. CVA is tolerant of both low and high TP levels, and is difficult to adapt to the environment above 3,920 m altitude. In terms of the change in CVA community structure, the environmental factors significantly affected by each genus are different, and the main environmental factors include Temp, pH, DO, TN, and TP. As the pollution level of the river system increases, the scale, density, and connectivity of CVA network would decrease, thus its diversity will be affected.

We investigated the ideal CVA distribution area using the MaxEnt model. The findings indicate that the main CVA distribution areas are Yunnan Province, the Three Gorges Reservoir Area, Hubei Province, Anhui Province, Jiangsu Province, and Shanghai. The Three Gorges Reservoir Area, Chaohu Lake, and Taihu Lake are still highly suitable areas for CVA even after future climate change. The distribution and biogeography of CVA will be severely impacted by temperature changes brought on by climate change. Under future climate change, the distribution fragmentation of high suitability areas for CVA may increase and need further investigation.

## Data availability statement

The datasets presented in this study can be found in online repositories. The names of the repository/repositories and accession number(s) can be found at: http://www.ncbi.nlm.nih.gov/bioproject/891953, PRJNA891953.

## Author contributions

YH: conceptualization, data curation, formal analysis, methodology, validation, visualization, writing—original draft, and writing—review and editing. JZ, JH, and MZ: sampling. SH: funding acquisition. All authors have read and agreed to the published version of the manuscript.

## Funding

This research was funded by National Key Research and Development Program of China, grant number 2021YFC3201002.

## Conflict of interest

The authors declare that the research was conducted in the absence of any commercial or financial relationships that could be construed as a potential conflict of interest.

## Publisher’s note

All claims expressed in this article are solely those of the authors and do not necessarily represent those of their affiliated organizations, or those of the publisher, the editors and the reviewers. Any product that may be evaluated in this article, or claim that may be made by its manufacturer, is not guaranteed or endorsed by the publisher.

## Supplementary material

The Supplementary material for this article can be found online at: https://www.frontiersin.org/articles/10.3389/fmicb.2023.1078081/full#supplementary-material

## Footnotes

^1^ www.ncbi.nlm.nih.gov/nuccore

^2^ https://blast.ncbi.nlm.nih.gov/Blast.cgi

^3^ https://www.worldclim.org/

^4^ https://www.worldclim.org/data/bioclim.html
