# Supplementary figures and images for: The biogeography of colonial volvocine algae in the Yangtze River basin
Source: Front Microbiol. 2023 Jan 26;14:1078081. doi: 10.3389/fmicb.2023.1078081 (PMC9910701; doi:10.3389/fmicb.2023.1078081)

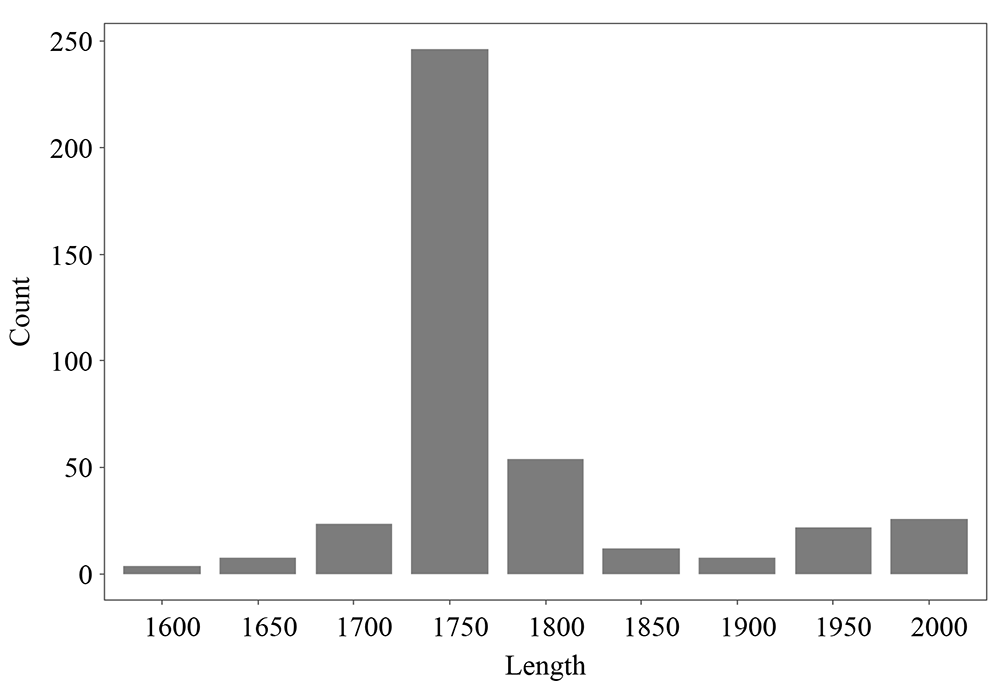

Supplement: Supplementary file 1 [file Image_1.TIF]

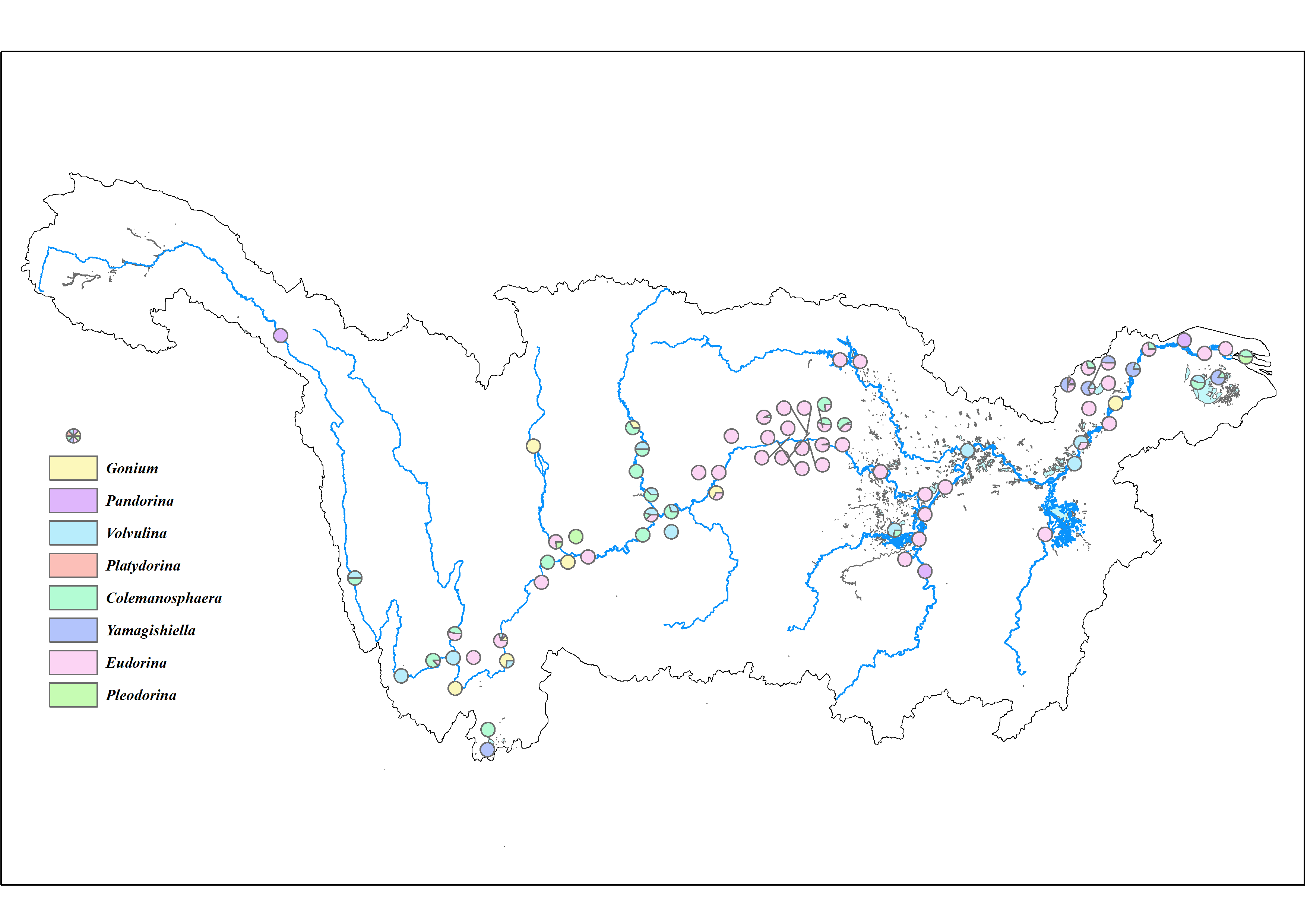

Supplement: Supplementary file 2 [file Image_2.TIF]
